# Mitigative efficacy of the clinical dosage administration of granulocyte colony-stimulating factor and romiplostim in mice with severe acute radiation syndrome

**DOI:** 10.1186/s13287-020-01861-x

**Published:** 2020-08-03

**Authors:** Masaru Yamaguchi, Marino Suzuki, Moeri Funaba, Akane Chiba, Ikuo Kashiwakura

**Affiliations:** Department of Radiation Science, Hirosaki University Graduate School of Health Sciences, 66-1 Hon-cho, Hirosaki, Aomori, 036-8564 Japan

**Keywords:** Acute radiation syndrome, Approved pharmaceutical drugs, Granulocyte colony-stimulating factor, Romiplostim, Radiation medical countermeasure

## Abstract

**Background:**

It has been reported that the high-dosage administration of domestically approved pharmaceutical drugs, especially granulocyte colony-stimulating factor (G-CSF) and romiplostim (RP), is a rapid and appropriate medical treatment for preventing severe acute radiation syndrome (ARS) of victims exposed to lethal total-body irradiation (TBI). However, it remains unclear whether or not the clinical dosage administration of these drugs can ameliorate TBI-induced ARS and related high mortality in order to find various drug treatment options and less toxic optimum protocol depending on the situation surrounding the radiological accidents.

**Methods:**

We assessed the clinical dosage administration in combination with G-CSF and RP as intraperitoneal injection in C57BL/6 J mice exposed to more than 7-Gy lethal dose of X-ray TBI for the survival study evaluated by the log-rank test. Bone marrow and splenic cells were collected on the 21st day, when 1 week has passed from last administration, to detect the level of cell apoptosis, intracellular reactive oxygen species (ROS), and nuclear factor erythroid 2-related factor 2 (Nrf2)-related anti-oxidative gene expressions, and enzyme-linked immune sorbent assay using sera was performed for cell senescence and inflammation status analyzed with one-way ANOVA and Tukey-Kramer or Bonferroni/Dunn multiple comparison tests.

**Results:**

The combined once-daily administration of 10 μg/kg G-CSF for 4 times and 10 μg/kg RP once a week for 3 times improve the 30-day survival rate of lethal TBI mice compared with untreated TBI mice, accompanied by a gradual increase in the body weight and hematopoietic cell numbers. The radio-mitigative effect is probably attributed to the scavenging of ROS and the reduction in cell apoptosis. These changes were associated with the upregulation of Nrf2 and its downstream anti-oxidative targets in TBI mice. Furthermore, this combination modulated TBI-induced cell senescence an d inflammation markers.

**Conclusions:**

This study suggested that the clinical dosage administration in combination with G-CSF and RP may also have radio-mitigative effects on mice exposed to lethal TBI and may be a potent therapeutic agent for mitigating radiation-induced severe ARS.

## Background

Total-body irradiation (TBI) exposure of greater than 1 Gy in the event of an unexpected radiological/nuclear accident results in well-understood acute adverse effects on the hematopoietic system due to this system’s strong radio-sensitivity. This dysfunction derived from acute radiation syndrome (ARS) is characterized by dose-dependent bone marrow destruction and, at worst, individual death within a few months [1]. Therefore, appropriate medical treatments should be performed immediately after TBI in order to achieve reconstitution and restoration of hematopoiesis and avoid ARS-induced mortality. Following an accidental or deliberate radiological scenario accompanied by a large number of victims, the intake of appropriate medications using stably supplied and regularly stockpiled approved pharmaceutical drugs is the most suitable initial treatment, as one should expect substantial delays in delivering good medical care to the affected population and bone marrow transplantation for the recovery from radiation-induced bone marrow damage of victims in radiation accidents has many limitations, including histocompatibility, age constraints, human leukocyte antigen type, and the fact that immunosuppression would be required to reduce the risk of graft versus host rejection. While efforts to identify and develop radiation medical countermeasures (MCMs) for ARS were first initiated decades ago [2, 3], various radio-protective/mitigative agents, such as thiol-containing compounds, antioxidants, growth factors/cytokines, and apoptosis inhibitors, have been designed and reported [4, 5] but some candidate drugs have a relatively high toxicity or are clinically inconsequential due to poor radio-protection [6]. There are only three US Food and Drug Administration-approved MCMs available that increase the survival in patients: two granulocyte colony-stimulating factors (G-CSFs) and the granulocyte macrophage colony-stimulating factor (GM-CSF) [7]. In Japan, four recombinant G-CSFs have been approved for the treatment of neutropenia, including cancer chemotherapy [8]. However, no effective action on the thrombocytopenia or excessive bleeding that occurs in ARS is expected. On the other hand, the combination of G-CSF and MCMs that can address thrombocytopenia can be expected to improve the pancytopenia occurs in ARS patients, since the duration of severe thrombocytopenia appears to correlate with death to a greater extent than that of severe neutropenia and appears to be more clinically relevant to the survival in ARS [9]. The approval of cytokine use varies by country, and GM-CSFs, interleukin-3, and thrombopoietin (TPO), which are recommended as the principal therapeutic cytokines corresponding to ARS severity by the International Atomic Energy Agency [10], are not approved in Japan. We recently found that treatment with a combination of approved pharmaceutical drugs, such as a G-CSF (Neutrogin^®^) and the TPO receptor (TPOR) agonist romiplostim (RP; Romiplate^®^), rescues mice from lethal TBI [11–13]. Although the effectiveness of these drugs as a medical treatment for emergency radiation exposure has been reported, the necessary dosages reported were much higher than the clinically used dosages. It would therefore be desirable to identify suitable drug treatment options with a less-toxic therapeutic protocol. In addition, effective MCMs with low toxicity for emergency radiation medical care are needed while considering the medical situation or regulations of therapeutic goods in each country. In the present study, to establish an optimum therapeutic protocol using domestically approved pharmaceutical drugs to increase the survival of TBI exposed victims, we assessed various combinations of clinical dosage administrations of G-CSF and RP in mice exposed to a lethal TBI. These findings could be expected to contribute to emergency radiation medical care and also reduction of side effects on normal tissues in radiotherapy.

## Methods

### Animal experiments

Seven-week-old female C57BL/6JJcl mice were delivered from the breeding facilities of CLEA Japan (Tokyo, Japan). All mice were housed in a conventional clean room at an ambient temperature of 23 °C, relative humidity of 50%, and 12-h light/dark cycle. The mice had ad libitum access to sterilized standard laboratory mouse chow diet (CLEA Rodent Diet CE-2, CLEA Japan) and drinking water. In the present study, the selection criteria applied prior to sacrifice were > 20% loss of body weight and respiratory distress. All experiments were conducted according to the legal regulations in Japan and the Guidelines for Animal Experiments after obtaining approval from the animal experimental committee (approved number: G17001).

### Lethal X-ray TBI in mice

After a week of acclimatization, 8-week-old mice were randomly subjected to varying lethal TBI doses of 7, 7.25, or 7.5 Gy of X-rays (150 kVp, 20 mA, 0.5-mm aluminum and 0.3-mm copper filters) at a dose rate of 1.0 Gy/min using an MBR-1520R X-ray generator (Hitachi Medical, Tokyo, Japan). Within 2 h after TBI, the mice were administered the medications described below. In addition to mice treated with TBI only (TBI mice), mice that received TBI and medication (TBI plus drug combination mice), those treated with medication only (drug combination mice), and those that did not receive either TBI or medication (control mice) were also evaluated in this study. The numbers of mice included in these experimental groups are indicated in the figure legends.

### Drug administration

The post-radiation treatment was started within 2 h after TBI. Drug combination mice with or without TBI were administered different types of medications (Table 1). The four types of medications were delivered in combinations of the following two commercially available drugs based on our previous reports [11–13]: recombinant human G-CSF (Neutrogin^®^; Chugai Pharmaceutical, Tokyo, Japan) and human TPOR agonist RP (Romiplate^®^; Kyowa Hakko Kirin, Tokyo, Japan). G-CSF and RP were intraperitoneally administered once-daily for 3 or 4 times and once a week for up to 4 times, respectively. The dose of G-CSF and RP was 10 μg/kg of body weight/day, which was the same as the clinically used dose [14–16]. Mice treated with TBI only and control mice alternatively received injections of normal saline solution (Otsuka Pharmaceutical, Tokyo, Japan) as the vehicle used to prepare the drugs. Peripheral blood was harvested from orbital venous plexus of mice anesthetized using isoflurane (Powerful Isoful; Zoetis, London, UK), and placed at room temperature for at least 30 min to allow blood-clotting. Sera were collected by centrifugation at 3000 rpm for 10 min. The separated serum samples were stored at − 80 °C until the analysis.

**Table 1:** The four combinations of medications subsequent to lethal TBI

Medication	Drugs	Intraperitoneally administration timing after TBI						Total frequency (times)
		< 2 h	1 day	2 days	7 days	14 days	21 days	
#1	G-CSF	→	→	→				3
	RP	→			→			2
#2	G-CSF	→	→	→				3
	RP	→			→	→		3
#3	G-CSF	→	→	→				3
	RP	→			→	→	→	4
#4	G-CSF	→	→	→	→			4
	RP	→			→	→		3

The right arrow means the timing of intraperitoneally administration in each combination

### Collection of bone marrow and splenic cells

Bone marrow cells (BMCs) were harvested from both femurs by flushing with ethylenediaminetetraacetic acid-phosphate buffered saline (PBS), pH 7.4, containing 0.5% bovine serum albumin using a 26-gauge needle. BMCs suspended in media were gently pipetted a few times. The spleen was weighted and placed in Hanks’ balanced salt solution. Using the ends of a spatula, the spleen was thoroughly dissociated by gently scrunching and pressing. After centrifuging at 1400 rpm for 10 min, the BMCs and splenic cells were treated with Gey’s salt solution for red blood cell lysis. After removal of the lysed red blood cells, the viable BMCs and splenic cells were filtered through a 45-μm strainer and then counted using a hemocytometer (BurkerTurk; Sunlead Glass, Saitama, Japan) with the trypan blue dye exclusion method (Sigma-Aldrich^®^, St. Louis, MO, USA).

### Cell death analyses

Apoptosis was analyzed using the fluorescein isothiocyanate (FITC) Annexin V apoptosis Detection Kit with propidium iodide (PI) (BioLegend, San Diego, CA, USA) according to the manufacturer’s instructions. In brief, BMCs and splenic cells were washed by PBS and suspended in Annexin V binding buffer. The Annexin V-FITC and 0.5 mg/ml PI solution were then added to the cell suspension, which was incubated for 15 min at room temperature in the dark. The cells were resuspended in Annexin V binding buffer and then analyzed by flow cytometry (FC500; Beckman Coulter^®^, Fullerton, CA, USA). In the Annexin V/PI quadrant gating, cells were classified as Annexin V/PI double-negative, Annexin V-positive PI-negative, or Annexin V/PI double-positive, representing viable, early apoptotic, and late apoptotic/necrotic cells, respectively.

### Measurement of intracellular reactive oxygen species (ROS) generation

The fluorescent probe 5-(and-6)-chloromethyl-2′,7′-dichlorodihydrofluorescein diacetate, acetyl ester (CM-H_2_DCFDA) (Thermo Fisher Scientific, Boston, MA, USA) was used for the assessment of intracellular ROS, such as hydroxyl radical, hydrogen peroxide, and peroxynitrite. BMCs and splenic cells were incubated for 20 min with 5 μM CM-H_2_DCFDA in PBS at 37 °C in a humidified atmosphere with 5% CO_2_. Unincorporated CM-H_2_DCFDA was removed by washing with PBS. Each sample was resuspended in PBS and analyzed by flow cytometry.

### Enzyme-linked immunosorbent assay (ELISA) analyses

The concentrations of plasminogen activator inhibitor (PAI-1), tumor necrosis factor α (TNF-α), and cyclin-dependent kinase inhibitor 2A (CDKN2A/p16^INK4a^) in the sera were measured with commercially available ELISA kits according to the manufacturers’ protocols. All samples subjected to these assays were quickly thawed serum. Each sample was individually placed in a well pre-coated with an antibody against the target protein and then incubated to allow binding of the antibodies immobilized at the bottom of the well. Subsequently, biotinylated antibodies for each of the antibodies and horseradish peroxidase-conjugated streptavidin were added and reacted according to the manufacturers’ protocols. After the 3-, 3′-, 5-, 5′-tetramethylbenzidine substrate addition and following stop solution reaction, the concentrations of the target protein were measured at an absorbance of 450 nm using a standard curve obtained from standard solutions. The ELISA kits for murine PAI-1, TNF-α, and CDKN2A/p16^INK4a^ were PAI-1 (SERPINE1) Mouse ELISA Kit (Thermo Fisher Scientific), Mouse TNF-alpha ELISA Kit (Protein Tech Japan, Tokyo), and Mouse CDKN2A ELISA kit (LifeSpan BioSciences, Seattle, WA, USA), respectively.

### Total RNA extraction

RNA, including miRNA, was extracted from BMCs and splenic cells using miRNeasy Mini Kit (QIAGEN, Hilden, Germany) according to the manufacturer’s instructions. 3 × 10^6^ cells were added to 0.7 ml of QIAzol Lysis Reagent, shaken vigorously, and incubated for 5 min at room temperature to promote a complete dissociation of nucleoprotein complexes. After adding 140 μl of chloroform to separate the aqueous and phenolic phases, the homogenate was vigorously shaken for 15 s and stored for 3 min at room temperature. After centrifuging at 12,000*g* for 15 min, total RNA was precipitated from the aqueous phase using 100% ethanol. The purification of total RNA was achieved using RNeasy mini columns according to the protocol provided by the manufacturer. The RNA was then eluted from the column by adding 30 μl of RNase-free water. The quality and concentration of the yielded RNA were assessed using a NanoDrop spectrophotometer (NanoDrop Technologies, Wilmington, DE, USA). All RNA samples had 260/280-nm absorbance ratios of 1.8–2.0.

### Quantitative reverse transcription polymerase chain reaction (qRT-PCR) analysis

First-strand complementary DNA from 100 ng RNA was synthesized using the SuperScript™ IV VILO™ Master Mix with ezDNase (Thermo Fisher Scientific) according to the manufacturer’s instructions. A total of 20 μl reaction solution was reacted with the following parameters: 25 °C for 10 min, 50 °C for 10 min, and then 85 °C for 5 min, in order to synthesize complementary DNA. qRT-PCR was performed using the Power SYBR^®^ Green Master Mix (Applied Biosystems, Carlsbad, CA, USA) and a StepOnePlus™ Real-Time PCR System (Thermo Fisher Scientific). ATPase subunit 6 mRNA (*ATP6*) was used as an internal control for all reactions because the fluctuation of *ATP6* was the lowest among 16 housekeeping genes and 16 mouse orthologs of human internal standard genes analyzed with the TaqMan^®^ Array Mouse Endogenous Control 96-well Plate (Thermo Fisher Scientific) in the preliminary test. We performed qPCR with the following typical amplification parameters: 95 °C for 10 min, followed by 40 cycles of 95 °C for 15 s and 60 °C for 1 min. Relative differences in the gene expression were determined by the *ΔΔ*CT method. The mRNA expression of control mice was defined as the baseline. The oligonucleotide primer sets used in this analysis of related nuclear factor erythroid 2-related factor 2 (Nrf2) target genes, such as heme oxygenase 1 (*Ho-1*), ferritin heavy polypeptide 1 (*Fth1*), NAD(P) H dehydrogenase quinone 1 (*Nqo1*), glutamate-cysteine ligase catalytic subunit (*Gclc*), glutamate-cysteine ligase modifier subunit (*Gclm*), glutathione reductase (*Gsr*), and thioredoxin reductase 1 (*Txnrd1*), and the internal control *ATP6* were purchased from Eurofins Genomics Inc. (Tokyo, Japan) (Table 2).

**Table 2:** Sequences of PCR primers

Gene	Primer sequence (5′ to 3′)
*Ho-1*	F: AGGGTCAGGTGTCCAGAGAA R: CTTCCAGGGCCGTGTAGATA
*Fth1*	F: TGGAGTTGTATGCCTCCTACG R: TGGAGAAAGTATTTGGCAAAGTT
*Nqo1*	F: AGCGTTCGGTATTACGATCC R: AGTACAATCAGGGCTCTTCTCG
*Gclc*	F: AGATGATAGAACACGGGAGGAG R: TGATCCTAAAGCGATTGTTCTTC
*Gclm*	F: TGACTCACAATGACCCGAAA R: TCAATGTCAGGGATGCTTTCT
*Gsr*	F: ACTATGACAACATCCCTACTGTGG R: CCCATACTTATGAACAGCTTCGT
*Txnrd1*	F: TCTGAAGAAAAAGCCGTAGAGAA R: TTCCAATGGCCAAAAGAAAC
*ATP6*	F: CCATAAATCTAAGTATAGCCATTCCAC R: AGCTTTTTAGTTTGTGTCGGAAG

*F* forward primer, *R* reverse primer

### Statistical analyses

Data are represented as the mean ± standard deviation (SD). The levels of significance were calculated using the Excel 2016 software program (Microsoft, Redmond, WA, USA) with the Statcel3 add-on (OMS, Saitama, Japan). Survival studies’ data were analyzed using the Kaplan-Meier method followed by the Mantel-Cox (log-rank) test for the assessment of significant differences. *P* values of < 0.01 or 0.05 were considered to indicate statistical significance by *t* test for the comparison between two groups. In addition, the data were analyzed with one-way ANOVA and Tukey-Kramer or Bonferroni/Dunn multiple comparison tests statistically significant. The statistical method used in each experiment is indicated in the figure legends.

## Results

### Survival rate following the clinical dosage administration in combination with approved pharmaceutical drugs

**Fig. 1:**
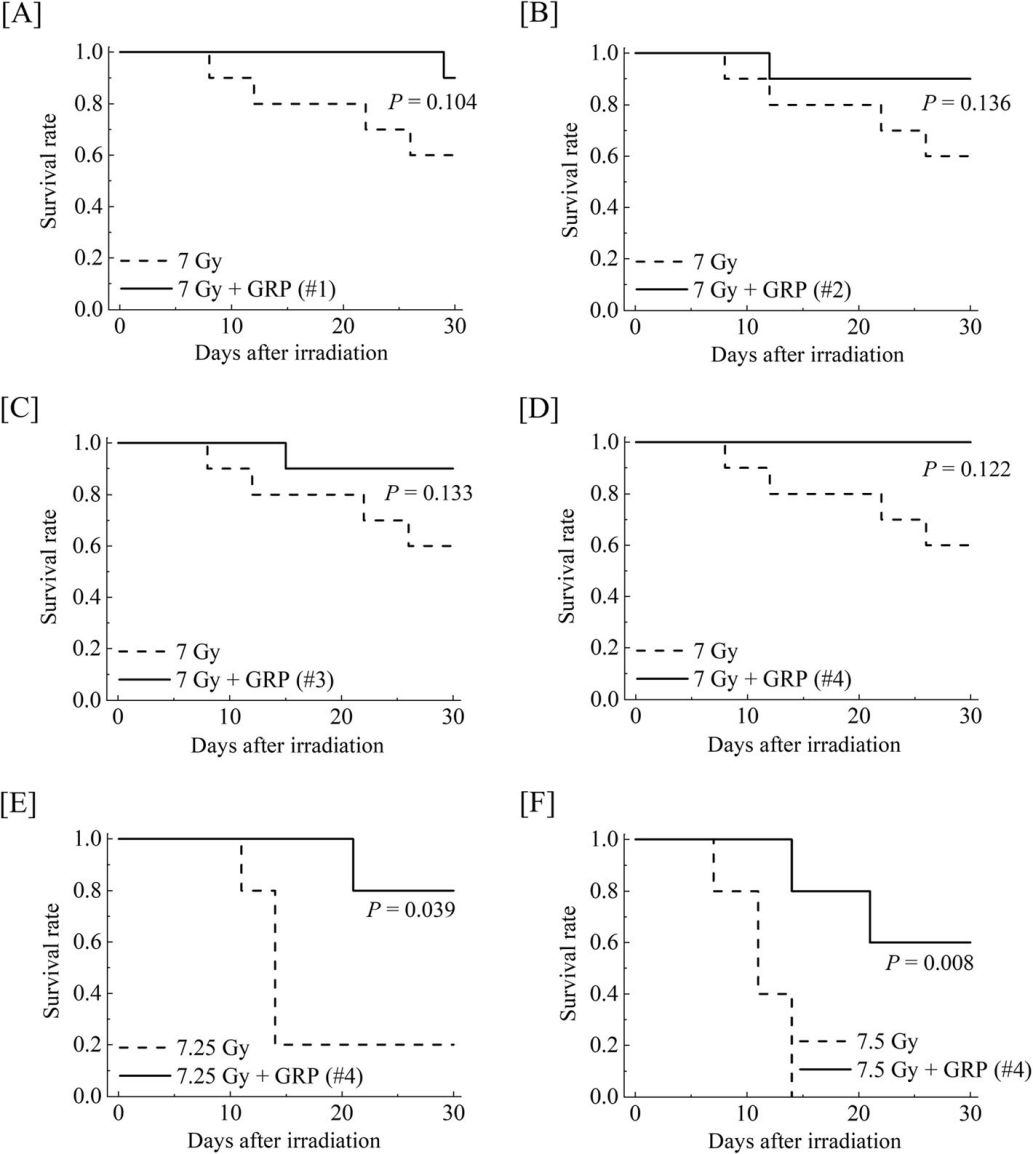
The Kaplan-Meier plots for the survival of mice treated with combinations of commercially available drugs. Mice were intraperitoneally administered a clinical dosage (10 μg/kg of body weight/day) of G-CSF and RP, starting within 2 h after 7-Gy dose of TBI. As shown in Table 1, the medications were administered in 4 different combinations (*n* = 10 in each group): **a** G-CSF once-daily for 3 times and RP once a week for 2 times (GRP #1), **b** G-CSF once-daily for 3 times and RP once a week for 3 times (GRP #2), **c** G-CSF once-daily for 3 times and RP once a week for 4 times (GRP #3), and **d** G-CSF once-daily for 4 times and RP once a week for 3 times (GRP #4). In addition, mice were intraperitoneally administered the GRP #4 starting within 2 h after **e** 7.25 Gy or **f** 7.5 Gy of TBI (*n* = 5 in each group). Mice treated with TBI only received injections of the normal saline solution as vehicle. “7 Gy + GRP,” “7.25 Gy + GRP,” or “7.5 Gy + GRP” and “7 Gy,” “7.25 Gy,” or “7.5 Gy” indicated the results of TBI mice with and without each medication, respectively. Statistically significant differences were evaluated by the log-rank test compared with TBI only (*P* < 0.05)

To determine the effects of the clinical dosage administration in combination with approved pharmaceutical drugs on the survival of mice exposed to high-dose TBI (7 Gy), 4 combinations of G-CSF and RP were administered to mice within 2 h after X-irradiation. The combinations and schedules are summarized in Table 1. The doses of G-CSF and RP used in the present study (10 μg/kg of body weight/day) were the same as the clinically used doses. The survival rates with each medication are shown in Fig. 1. Mice exposed to the 7-Gy dose of TBI alone showed a 60% 30-day survival rate. In contrast, although no statistically significant difference was observed between any drug combination mice with or without TBI, all combinations improved the 30-day survival rate to over 90% (Fig. 1a–d). Among them, combination #4 (once-daily G-CSF for 4 times and RP once a week for 3 times) especially showed a complete 30-day survival rate (Fig. 1d). In order to evaluate the relationship between the lethal effect of a more than 7-Gy dose of TBI and the radio-mitigative effect of combination #4, G-CSF and RP were administered as per the protocol after TBI, and the survival was monitored for up to 30 days. When exposed to lethal doses of TBI (7.25 Gy), the 30-day survival rate of TBI mice was 20% (Fig. 1e). The administration of combination #4 significantly improved the survival rate to 80% (*P* < 0.05). In addition, while a 7.5-Gy dose of TBI was fatal in all TBI mice within 14 days, post-exposure treatment with combination #4 resulted in a 60% survival rate at 30 days (*P* < 0.01, Fig. 1f). These results indicated that combination #4 was the most suitable medication for improving the 30-day survival rate and subsequent studies were performed at a radiation dose of 7.25 Gy.

### Mitigation of lethal TBI-induced hematopoietic damages by pharmaceutical drugs

**Fig. 2:**
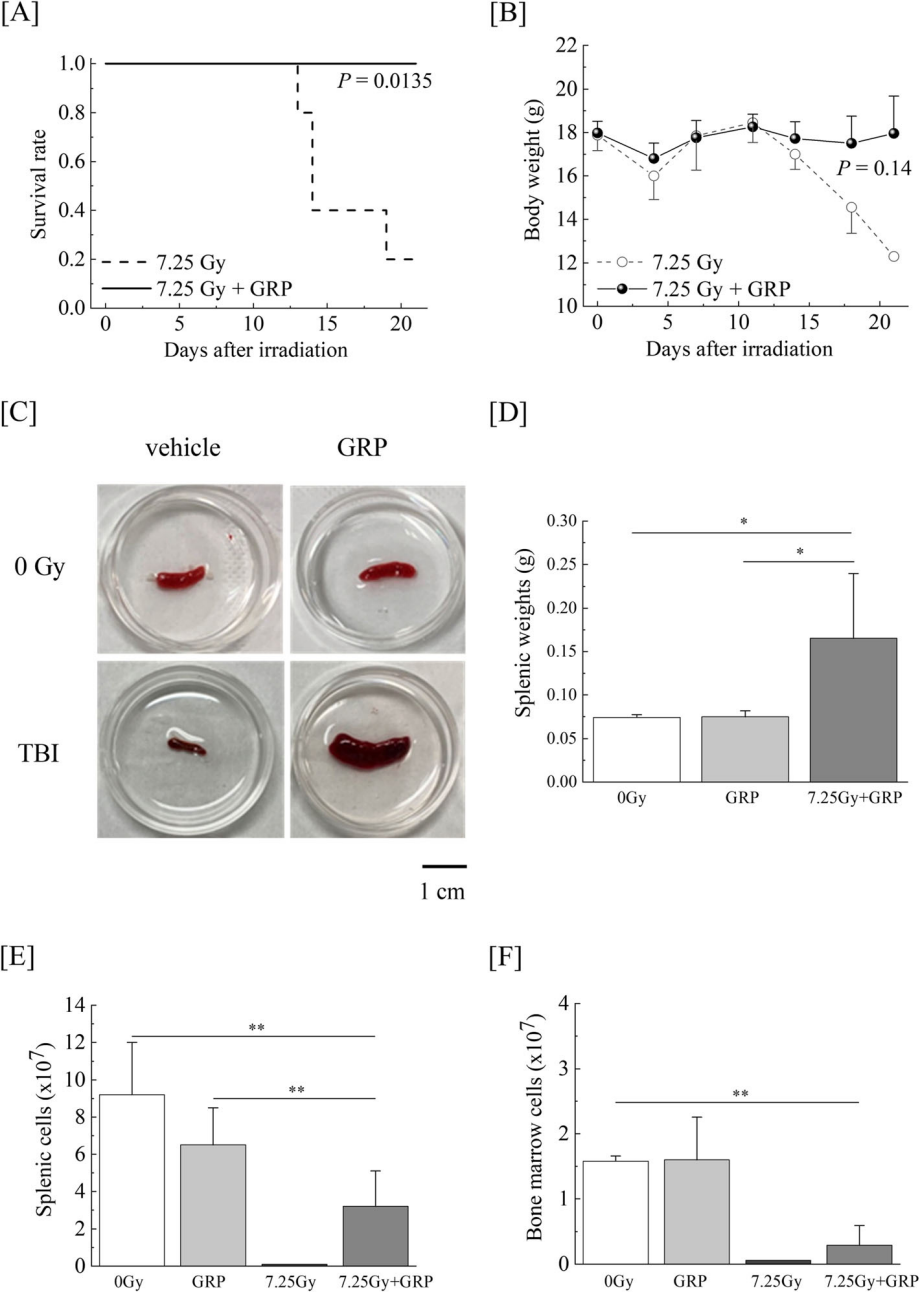
Commercially available drugs attenuate lethal TBI-induced hematopoietic reduction. Mice were intraperitoneally administered the clinical dosage (10 μg/kg of body weight/day) of G-CSF once-daily for 4 times and RP once a week for 3 times (GRP #4) starting within 2 h after exposure to a 7.25-Gy dose of TBI. Mice treated with TBI only received injections of the normal saline solution as vehicle. “7.25 Gy + GRP” and “7.25 Gy” indicate the results of TBI mice with and without GRP #4, respectively. **a** Mitigative effects of GRP #4 administered immediately post-TBI are represented in a Kaplan-Meier survival curve until day 21 (*n* = 5 in each group). Statistically significant difference was evaluated by the log-rank test compared with TBI only (*P* < 0.05). **b** Body weight changes in the surviving mice (*n* = 5 in each group). **c** Representative photographs of splenic endogenous colonies on day 21 are shown. Scale bars, 1 cm. **d** Splenic weights and total viable cell numbers in the **e** spleen and **f** bone marrow observed in the TBI and control mice treated with or without GRP #4 (*n* = 5 per group). The data are expressed as the means ± SD. Statistically significant differences were evaluated by one-way ANOVA and the multiple comparison tests (**P < 0.01, *P < 0.05)

To assess the radio-mitigative effects of approved pharmaceutical drugs against a lethal 7.25-Gy dose of TBI-induced hematopoietic injury, the bone marrow and spleen of TBI mice treated with or without combination #4 were examined. At day 21 post-TBI, when 1 week has passed from last administration, the survival rate of TBI mice was approximately 20%, whereas all TBI mice survived following the administration of combination #4 (*P* = 0.0135, Fig. 2a). While TBI mice treated with or without combination #4 showed a similar growth until day 12, the body weights of the TBI mice which received combination #4 were higher than those of the TBI mice on day 21, although not to a significant degree (*P* = 0.14, Fig. 2b). At that time, the femurs and spleen were collected from the surviving mice. A significant decrease in the splenic size was observed in the TBI mice (Fig. 2c). However, in the TBI mice treated with combination #4, splenic atrophy was not apparent and the splenic size on day 21 was 2-fold higher than that in the control mice. The splenic weight was also found to be markedly reduced in the TBI mice (data not shown), but this weight was drastically higher than that in the control mice following medication (Fig. 2d). Splenic endogenous colonies serve as an indicator of hematopoiesis, which is a crucial and indispensable factor dictating hematopoietic recovery post-TBI and, consequently, the survival [17, 18]. In addition, the number of viable splenic cells was significantly decreased after TBI and remained low until day 21 (Fig. 2e). The recovery of the decreased number of viable splenic cells occurred following drug combination #4 administration. In contrast, however, while the number of viable BMCs in the TBI mice treated with combination #4 gradually increased compared to the TBI mice, the counts remained nearly sixfold lower than the counts in the control mice on day 21, suggesting incomplete recovery (Fig. 2f). These results suggest that hematopoietic stresses mobilize hematopoietic stem/progenitors from the bone marrow to the spleen and induce extramedullary hematopoiesis [19, 20], and the administration of combination #4 to mice exposed to lethal TBI may enhance/recover the hematopoietic function in the spleen.

### Reduction in apoptotic cell death by pharmaceutical drugs

**Fig. 3:**
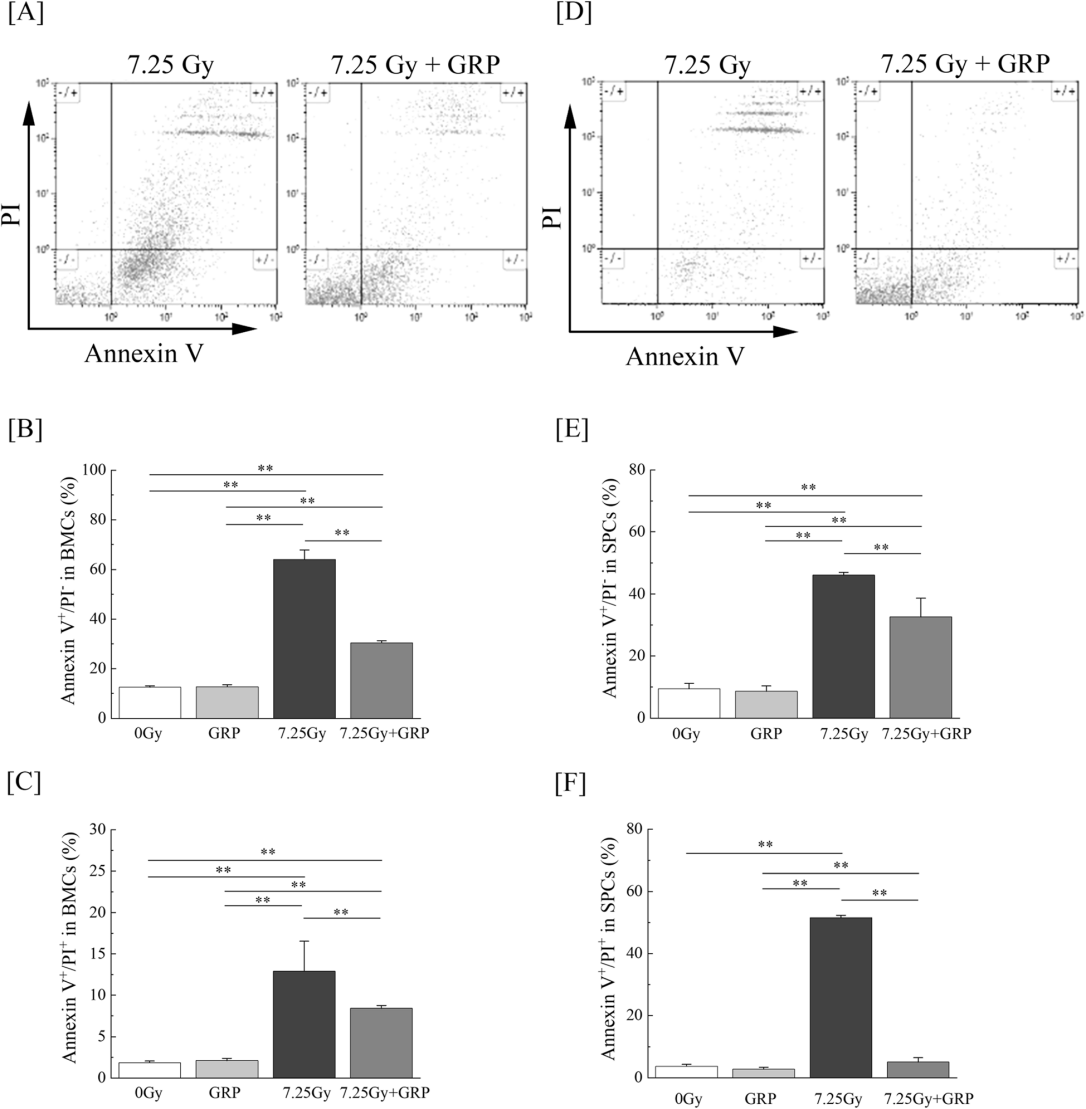
The inhibition of TBI-induced apoptotic cell death by commercially available drugs. Mice were intraperitoneally administered the clinical dosage (10 μg/kg of body weight/day) of G-CSF once-daily for 4 times and RP once a week for 3 times (GRP #4) starting within 2 h after a 7.25-Gy dose of TBI. Mice treated with TBI only received injections of the normal saline solution as vehicle. “7.25 Gy + GRP” and “7.25 Gy” indicate the results of TBI mice with and without GRP #4, respectively. **a** Representative flow cytometry plots of Annexin V/PI stained populations in BMCs. **b** The number of Annexin V-positive PI-negative populations in the bone marrow (*n* = 5 in each group). **c** The number of Annexin V/PI double-positive populations in the bone marrow (*n* = 5 in each group). **d** Representative flow cytometry plots of Annexin V/PI stained populations in splenic cells (SPCs). **e** The number of Annexin V-positive PI-negative populations in the spleen (*n* = 5 in each group). **f** The number of Annexin V/PI double-positive populations in the spleen (*n* = 5 in each group). The data are expressed as the means ± SD. Statistically significant differences were evaluated by one-way ANOVA and the multiple comparison tests (**P < 0.01)

Since TBI may induce irreparable DNA damage that can lead to disordered cell growth and cell apoptosis [21], a flow cytometric analysis of Annexin V/PI quadrant gating of apoptotic cell death was performed to assess the effects of clinical dosage administration of approved pharmaceutical drugs on the BMC and splenic cell survival on day 21. In Fig. 3a, d, cells were classified as Annexin V/PI double-negative, Annexin V-positive PI-negative, or Annexin V/PI double-positive, representing viable, early apoptotic, and late apoptotic/necrotic cells, respectively. We decided the Annexin V/PI double-negative gating as the fraction of viable cells using BMCs and splenic cells obtained from control mice (data not shown). The percentage of early apoptotic BMCs was significantly increased by TBI, but the drug combination #4 administration markedly reduced this trend by twofold compared to that in the TBI mice (Fig. 3b). The percentage of late apoptotic/necrotic BMCs also increased remarkably with TBI and tended to be significantly suppressed by combination #4 (Fig. 3c). In contrast, there was a notable increase in the proportions of early apoptotic and late apoptotic/necrotic splenic cells in the TBI mice (46.1 ± 0.8 and 51.5 ± 0.8, respectively). The combination #4 significantly reduced apoptotic cell death in both populations (32.6 ± 6.0 and 5.0 ± 1.4, respectively) (*P* < 0.01, Fig. 3e, f). Regarding the population of late apoptotic/necrotic splenic cells in particular, medications significantly suppressed the counts to nearly 90% of those observed in the TBI mice, leading to the attainment of baseline control levels. These findings show that the administration of combination #4 was able to prevent TBI-induced apoptosis of hematopoietic cells in vivo and contribute to the alleviation of hematopoietic failure.

### Scavenging of TBI-induced intracellular ROS by pharmaceutical drugs

**Fig. 4:**
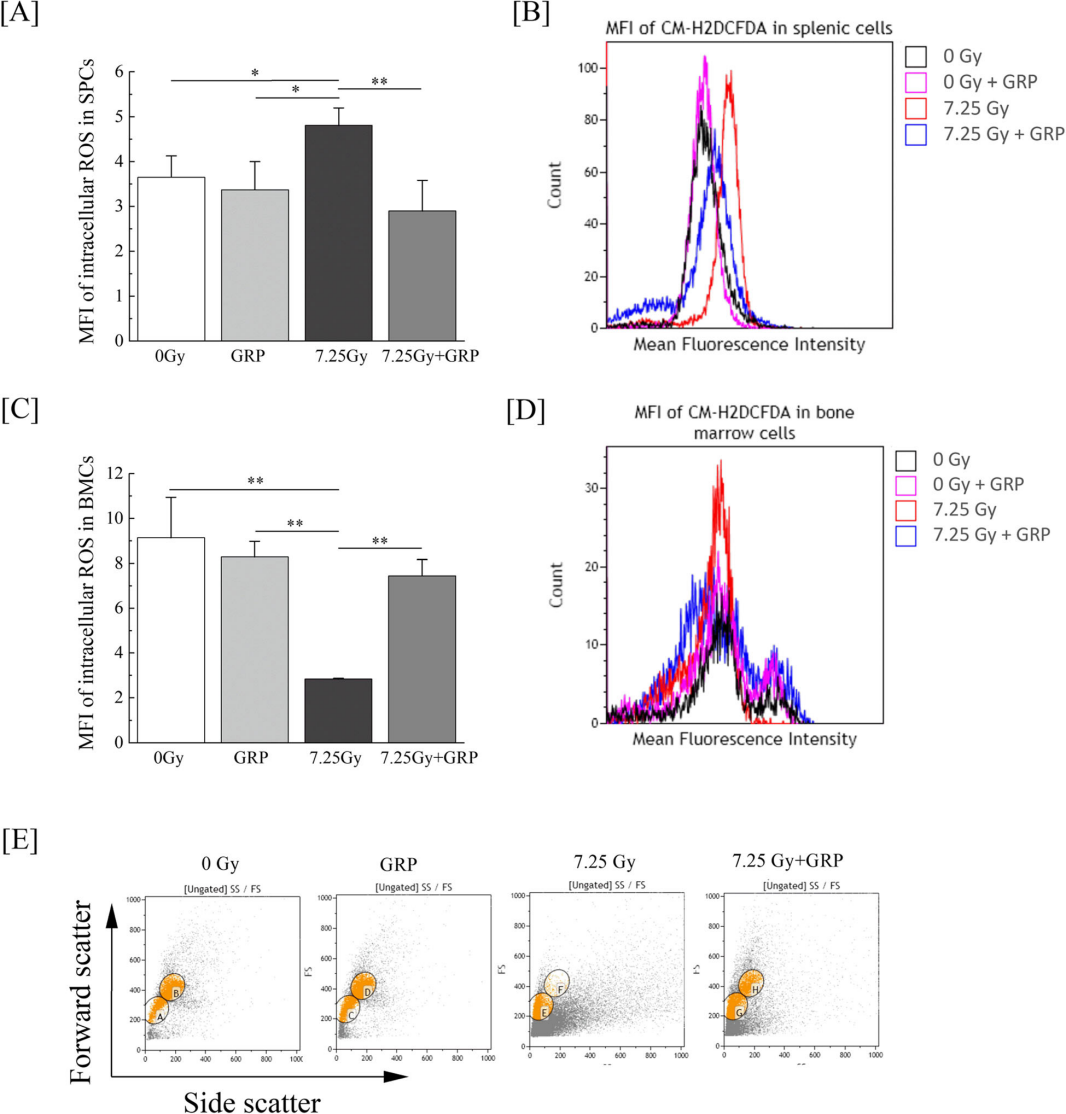
The alleviation of TBI-induced intracellular ROS generation by commercially available drugs. Mice were intraperitoneally administered the clinical dosage (10 μg/kg of body weight/day) of G-CSF once-daily for 4 times and RP once a week for 3 times (GRP #4) starting within 2 h after a 7.25-Gy dose of TBI. Mice treated with TBI only received injections of the normal saline solution as vehicle. “7.25 Gy + GRP” and “7.25 Gy” indicate the results of TBI mice with and without GRP #4, respectively. **a** The levels of ROS in splenic cells (SPCs) detected by the CM-H_2_DCFDA mean fluorescence intensity (MFI) (*n* = 5 in each group) and **b** the representative histogram of ROS levels by flow cytometry. **c** The levels of ROS in BMCs detected by the CM-H_2_DCFDA MFI (*n* = 5 in each group) and **d** the representative histogram of ROS levels by flow cytometry. **e** The representative flow cytometry plots of forward and side scatter of BMCs are shown. The data are expressed as the means ± SD. Statistically significant differences were evaluated by one-way ANOVA and the multiple comparison tests (**P < 0.01, *P < 0.05)

TBI may induce apoptotic cell death, resulting in myelosuppression, partly via the induction of oxidative stress in hematopoietic systems [22]. We examined intracellular ROS generation in response to TBI. ROS generation in splenic cells and BMCs after TBI at day 21 was analyzed by flow cytometry using CM-H_2_DCFDA staining, which can be detected by reacting with intracellular ROS, such as hydroxyl radicals, hydrogen peroxide, and peroxynitrite. As shown in Fig. 4a, b, the sustained generation of intracellular ROS in splenic cells was significantly elevated at day 21 after TBI compared to control mice. Treatment with combination #4 markedly attenuated the elevation of ROS production in the spleen, suggesting that this medication can effectively scavenge TBI-induced ROS production, especially hydroxyl radicals, hydrogen peroxide, and peroxynitrite, while in BMCs, the amount of ROS in the TBI mice was significantly decreased compared to other groups (Fig. 4c). According to the representative histogram of ROS production in BMCs in Fig. 4d, because a small peak was found to the right of the large peak in the control mice (black waveform) and drug combination mice with or without TBI (blue or purple waveform) but was not observed in the TBI mice (red waveform), the mean fluorescence intensity of ROS in the TBI mice was significantly decreased compared to other groups. More-differentiated and mature cells (corresponding to gate F) were presumably almost depleted in the TBI mice (Fig. 4e). These results indicate that the administration of combination #4 acts as an inhibitor (antioxidant) of TBI-mediated ROS generation.

### Regulation of the Nrf2-mediated antioxidant defense system by pharmaceutical drugs

**Fig. 5:**
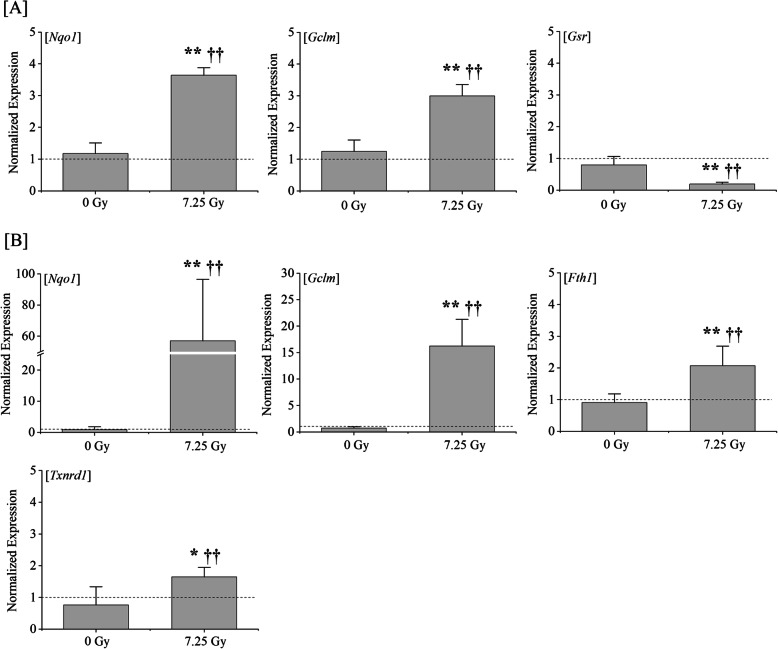
The regulation of the expression of Nrf2 signaling pathway’s downstream genes. Mice were intraperitoneally administered a clinical dosage (10 μg/kg of body weight/day) of G-CSF once-daily for 4 times and RP once a week for 3 times (GRP #4) starting within 2 h after a 7.25-Gy dose of TBI. Mice treated with TBI only received injections of the normal saline solution as vehicle. “7.25 Gy” and “0 Gy” indicate the results of GRP #4 treated mice with and without TBI, respectively. The relative quantitative mRNA expression of **a***Nqo1*, *Gclm*, and *Gsr* in BMCs and **b***Nqo1*, *Gclm*, *Fth1*, and *Txnrd1* in splenic cells, respectively, analyzed by qRT-PCR is presented as the means ± SD of the fold change (bar graph) compared with control mice (dotted line) (*n* = 5 in each group). *ATP6* was used as an internal control for all reactions. Statistically significant differences were evaluated by one-way ANOVA and the multiple comparison tests (**P < 0.01 and *P < 0.05 against control, or ^††^*P* < 0.01 and ^†^*P* < 0.05 against drug combination mice without TBI)

Nrf2, a central regulator of the endogenous antioxidant defense, has been implicated in the response to redox homeostasis [23]. To determine the underlying mechanisms by which the drug combination #4 protected TBI mice against oxidative stress, we evaluated the expression of the Kelch-like ECH-associated protein 1 (Keap1)/Nrf2 signaling pathway’s downstream genes, such as *Ho-1*, *Fth1*, *Nqo1*, *Gclc*, *Gclm*, *Gsr*, and *Txnrd1*, involved in redox reactions in BMCs and splenic cells by qRT-PCR. *ATP6* was used as an internal control for all reactions because the fluctuation of *ATP6* was the lowest among 16 housekeeping genes and 16 mouse orthologs of human internal standard genes analyzed with the TaqMan^®^ Array Mouse Endogenous Control 96-well Plate (data not shown). The mRNA expression of control mice was defined as the baseline and normalized to calculate the relative differences. The expression of *Nqo1* (*P* < 0.01) and *Gclm* (*P* < 0.01) was significantly increased on day 21 in BMCs of TBI mice treated with combination #4 compared to control mice (Fig. 5a). However, the expression of *Gsr* decreased significantly (*P* < 0.01). In contrast, a qRT-PCR analysis revealed that the administration of combination #4 resulted in a marked increase in the *Fth1* (*P* < 0.01), *Txnrd1* (*P* < 0.05), and especially *Nqo1* (*P* < 0.01) and *Gclm* (*P* < 0.01) expression in splenic cells at 21 days after TBI compared with the levels in control mice (Fig. 5b). These observations suggest that this medication reduces TBI-induced oxidative stress, possibly by regulating the Keap1/Nrf2 signaling pathway. Regarding Nrf2 target genes, such as *Ho-1* and *Gclc*, no significant differences were observed in BMCs and splenic cells in this study.

### Evaluation of the inflammatory/senescence profiles induced by TBI

**Fig. 6:**
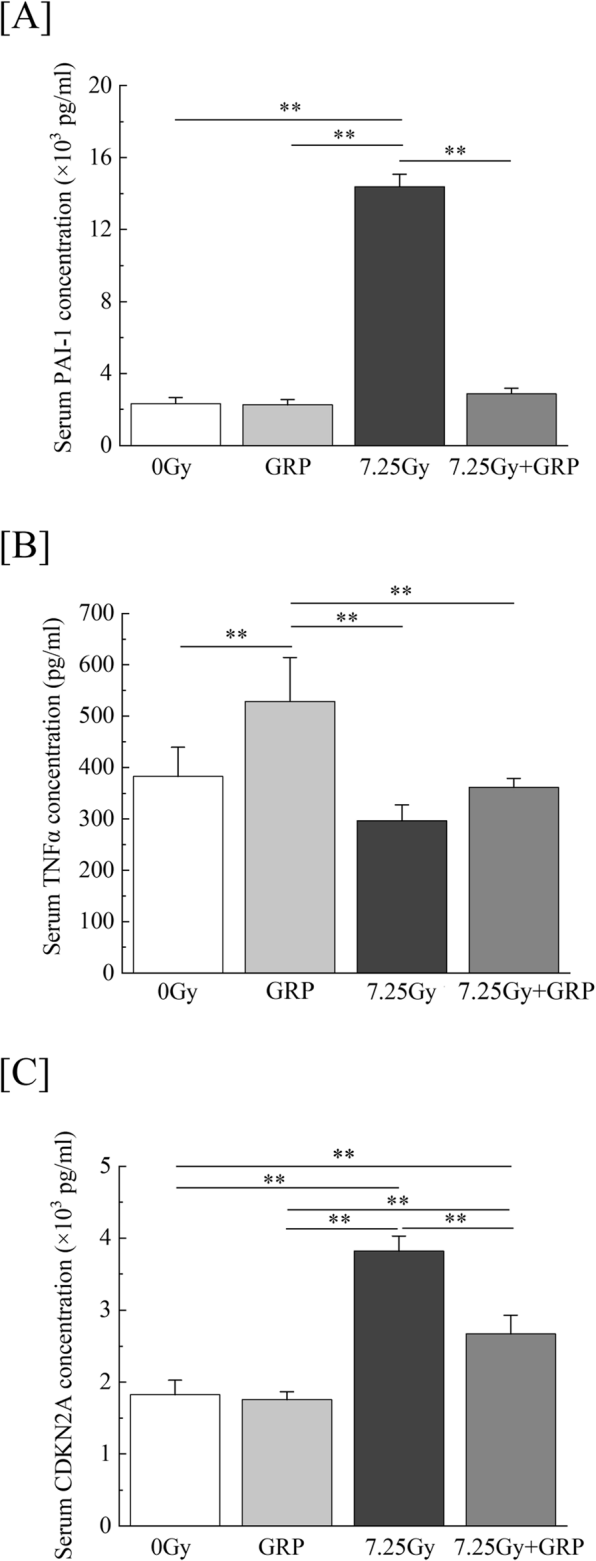
The effects of commercially available drugs on inflammation and cell senescence markers in serum. Mice were intraperitoneally administered a clinical dosage (10 μg/kg of body weight/day) of G-CSF once-daily for 4 times and RP once a week for 3 times (GRP #4) starting within 2 h after a 7.25-Gy dose of TBI. Mice treated with TBI only received injections of the normal saline solution as vehicle. “7.25 Gy + GRP” and “7.25 Gy” indicate the results of TBI mice with and without GRP #4, respectively. Peripheral blood was harvested from the orbital venous plexus of mice anesthetized using isoflurane, and serum samples were separated. The serum inflammation markers **a** PAI-1 and **b** TNF-α and the aging marker **c** CDKN2A/p16^INK4a^ were analyzed by an ELISA (*n* = 5 in each group). The data are expressed as the means ± SD. Statistically significant differences were evaluated by one-way ANOVA and the multiple comparison tests (**P < 0.01)

Since ionizing radiation has been reported to cause inflammation and cell senescence [24, 25], the combination #4 on TBI-induced inflammation markers PAI-1 and TNF-α and cellular senescence marker CDKN2A/p16^INK4a^ were measured using commercially available ELISA kits. The concentration of serum PAI-1 increased 7-fold in TBI mice compared to control mice on day 21, but the drug treatment with combination #4 suppressed it to the same extent as control mice (Fig. 6a). The serum concentration of TNF-α were found to be significantly enhanced by the administration of combination #4 compared to control mice but not increased in TBI mice (Fig. 6b). The serum CDKN2A/p16^INK4a^ levels, a biomarker of cell senescence, were significantly increased by TBI and significantly suppressed by combination #4 administration (Fig. 6c). However, this concentration in the TBI mice treated with medication was still higher than that in control mice on day 21. These results suggest that the administration of combination #4 suppresses some of the inflammation and cellular senescence that occurs in lethal TBI mice.

## Discussion

TBI exposure from radiotherapy or a radiological/nuclear accident results in adverse effects on cell/tissue structures and functions [26, 27]. Hematopoietic tissues are the most sensitive to radiation, and the rapid depletion of peripheral blood cells is a hallmark of hematopoietic disorders in ARS, which can ultimately lead to individual death. Various MCMs for ARS, such as thiol-containing compounds, cytokines, growth factors, and inhibitors of apoptosis, have been designed and reported [4, 5], but some candidate drugs have a relatively high toxicity and are clinically inconsequential due to poor radio-protection [6]. We previously reported that treatment with the combination of domestically approved pharmaceutical drugs (e.g., G-CSF and RP) enhances the survival of mice exposed to lethal TBI [11–13]. Although the effectiveness of these drugs as a medical treatment for emergency radiation exposure has been known, the necessary dosages reported were much higher than the clinically used dosages, and it would be desirable to establish suitable drug treatment protocols at clinical doses that can be immediately addressed, depending on the situation surrounding the radiological accidents. Based on the 30-day survival rate in this study, the clinical dosage administration of G-CSF once-daily for 4 times and RP once a week for 3 times (combination #4, Table 1) were shown to completely rescue mice from 7-Gy TBI (Fig. 1d). Furthermore, this medication drastically improved the 30-day survival rate of mice exposed to lethal 7.25- or 7.5-Gy TBI (80%, Fig. 1e, or 60%, Fig. 1f, respectively) compared to the TBI mice, suggesting that the clinical dosage administration in combination with G-CSF and RP may have radio-mitigative effects on lethal TBI mice and can be used as a potent therapeutic agent to mitigate radiation-induced severe ARS.

The administration of combination #4 was confirmed to increase the splenic weight (Fig. 2d) and the appearance of endogenous splenic colonies (Fig. 2c), and recovery in viable splenic cells was also observed (Fig. 2e). However, the number of viable BMCs in the TBI mice treated with combination #4 was still lower than in the control mice, suggesting an incomplete recovery (Fig. 2f). The spleen in mice, unlike humans, is a major hematopoietic tissue, and the increase in the splenic weight is correlated with the hematopoietic function in the spleen [28]. Hematopoietic stresses are well known to mobilize hematopoietic stem cells from the bone marrow to the spleen, which induces extramedullary hematopoiesis [19]. Bykov et al. also reported that lignin-derived polyphenolic composition with ammonium molybdate improved the 30-day survival rate and increased the formation of endogenous splenic colony-forming units, indicating that the radio-mitigative effects are mediated by the enhancement of extramedullary hematopoiesis in the spleen [29]. It is therefore possible that the administration of combination #4 also enhanced the hematopoietic function in the spleen instead of the damaged bone marrow of mice exposed to lethal TBI.

Low-linear energy transfer ionizing radiation generates ROS that can lead to biological damage and alter cellular signaling pathways involved in cell cycle arrest, DNA damage, and cell death apoptosis [30, 31]. On day 21 after TBI, the sustained generation of intracellular ROS in splenic cells was significantly elevated compared to control mice (Fig. 4a, b). The administration of combination #4 markedly attenuated the elevation of ROS production in the spleen. Regarding BMCs, the amount of ROS in the TBI mice treated with combination #4 was equivalent to the levels in control mice (Fig. 4c, d). Furthermore, cell death apoptosis was significantly increased in both BMCs (Fig. 3a–c) and splenic cells (Fig. 3d–f) but suppressed by the administration of combination #4. Recently, Vlachodimitropoulou et al. reported that eltrombopag, which is a small molecule oral TPOR agonist, is a powerful iron chelator that mobilizes iron and ferritin and reduces ROS [32], and TPO, which is an important and non-redundant cytokine for hematopoietic stem cell maintenance and expansion, also exerts a protective effect on iron-overload induced apoptosis by inhibiting oxidative stress and suppressing the mitochondrial pathways in cardiomyocytes [33]. In addition, TPOR agonists have been reported to support DNA repair in hematopoietic stem/progenitor cell populations by modulating the efficiency of the DNA-dependent protein kinase catalytic subunit-dependent non-homologous end-joining pathway [34, 35]. On day 21 after TBI, the foci of γ-H2AX, is the first step in recruiting and localizing DNA repair proteins [36], in BMCs and splenic cells of TBI mice treated with administration of combination #4 were undetectable and comparable to the control mice (data not shown). Consistent with previous studies, it was suggested that the administration of combination #4 had various influences acting on the ROS scavenging, DNA repair promoting, and subsequentially suppressing apoptosis in mice exposed to lethal TBI.

The mRNA expression of *Nqo1* and *Gclm* in BMCs (Fig. 5a) and *Nqo1*, *Gclm*, *Fth1*, and *Txnrd1* (Fig. 5b) in splenic cells was upregulated in TBI mice treated with combination #4 compared with control mice, which may be involved in the mitigative effects on the radiation damage in hematopoietic organs. Among the genes with an increased expression, *Nqo1* and *Gclm* in particular showed a marked increase in both BMCs and splenic cells at 21 days after TBI compared with the levels in control mice. *Nqo1* is an important enzyme in the human antioxidant defense system and is known to protect cell/tissues from various cytotoxic quinones and oxidative stress [37, 38]. *Gclm* forms a dimer with *Gclc* and becomes glutamate-cysteine ligase, a rate-limiting enzyme that produces glutathione, which has an antioxidant effect [39]. Ma et al. reported that ferulic acid protects human umbilical vein endothelial cells from radiation-induced oxidative stress by increasing the mRNA of antioxidant-related genes, such as *GCLM* and *NQO1*, in radiated cells via the phosphatidylinositol 3-kinase and extracellular signal-regulated kinase pathways [40]. In addition, epicatechin mitigates radiation-induced intestinal injury and promotes intestinal regeneration by suppressing oxidative stress through the promotion of Nrf2 translocation from the cytoplasm to nucleus, which activates the expression of *NQO1* [41]. Furthermore, theaflavin, a polyphenolic compound from black tea, has the potential to be used as a radio-protective agent to ameliorate TBI-induced hematopoietic injury [42]. These effects of theaflavin were associated with a decline in ROS levels and DNA damage in irradiated hematopoietic stem cells, and oxidative stress was reduced mainly by upregulating Nrf2 and its downstream targets *Nqo1*. Therefore, the administration of combination #4 to mice exposed to lethal TBI enhanced the intrinsic antioxidant systems, resulting in an increase in the splenic weight and viable cell counts, the appearance of endogenous colonies, and the suppression of apoptosis, which can help prevent mortality of mice exposed to lethal TBI. In contrast, the expression of *Gsr* in the BMCs of TBI mice treated with combination #4 was significantly decreased compared to that of control mice (Fig. 5a). The expression of Nrf2 target genes may be temporarily reduced because the half-life of Nrf2 is 20 min [43], or the Nrf2 target genes activated by the administration of combination #4 may differ between the spleen and bone marrow.

The present study also demonstrated the increase of inflammatory marker PAI-1 (Fig. 6a) and the cell senescence marker CDKN2A/p16^INK4a^ (Fig. 6c), and the upregulation of both was significantly suppressed by combination #4. PAI-1 is an important regulator of cellular senescence, and its inhibition produces anti-oxidative enzymes and suppresses ROS production/ROS-induced aging marker p16^INK4a^ expression, leading to the suppression of cell senescence in endothelial cells [44, 45]. The serum PAI-1 and CDKN2A/p16^INK4a^ levels were drastically downregulated in this study, suggesting that the administration of combination #4 might induce not only the reduction of TBI-induced vascular endothelial damage but also the suppression of cell/individual senescence.

This study revealed a significant improvement in the 30-day survival rate of mice exposed to lethal TBI following the clinical dosage administration in combination with the approved pharmaceutical drugs G-CSF and RP. These may function as effective radio-mitigative agents by activating the Keap1/Nrf2-dependent anti-oxidative response. TBI-induced severe oxidative stress ultimately induces cell death, which is involved in various disease states. The combination of pharmaceutical drugs used in this study may be able to be applied as an effective radio-mitigative agent to reduce severe oxidative stress induced by TBI and is expected to contribute to emergency radiation medical care and the reduction of side effects on normal tissues in radiotherapy. However, additional preclinical studies in various mouse strains and large mammalian species should be performed to firmly establish the mechanism of action and for the development of an optimal therapeutic protocol for emergency radiation medical care for humans in the future. As long as the threat of nuclear disaster exists, preparedness for effective drug therapy is extremely important as a risk management measure in a safe society.

## Conclusions

The clinical dosage administration in combination with the approved pharmaceutical drugs G-CSF and RP showed an effective radio-mitigative ability for mice exposed to lethal TBI and can therefore be used as a potent therapeutic agent to mitigate radiation-induced severe hematopoietic ARS.

## Data Availability

The datasets used and/or analyzed during the current study are available from the corresponding author on reasonable request.

## Abbreviations

ARS: Acute radiation syndrome
ATP6: ATPase subunit 6 mRNA
BMCs: Bone marrow cells
CDKN2A/p16^INK4a^: Cyclin-dependent kinase inhibitor 2A
CM-H_2_DCFDA: 5-(and-6)-Chloromethyl-2′,7′-dichlorodihydrofluorescein diacetate acetyl ester
FITC: Fluorescein isothiocyanate
Fth1: Ferritin heavy polypeptide 1
Gclc: Glutamate-cysteine ligase catalytic subunit
Gclm: Glutamate-cysteine ligase modifier subunit
G-CSF: Granulocyte colony-stimulating factor
GM-CSF: Granulocyte macrophage colony-stimulating factor
Gsr: Glutathione reductase
Ho-1: Heme oxygenase 1
Keap1: Kelch-like ECH-associated protein 1
MCMs: Medical countermeasures
Nqo1: NAD(P) H dehydrogenase quinone 1
Nrf2: Nuclear factor erythroid 2-related factor 2
PAI-1: Plasminogen activator inhibitor-1
PI: Propidium iodide
qRT-PCR: Quantitative reverse transcription polymerase chain reaction
ROS: Reactive oxygen species
RP: Romiplostim
SD: Standard deviation
SPCs: Splenic cells
TBI: Total-body irradiation
TNF-α: Tumor necrosis factor α
TPO: Thrombopoietin
TPOR: TPO receptor
Txnrd1: Thioredoxin reductase 1
